# Axial Compressive Behavior of Geopolymer Recycled Lump Concrete

**DOI:** 10.3390/ma13030533

**Published:** 2020-01-22

**Authors:** Haiyan Zhang, Jiancheng Liu, Bo Wu, Zhijian Zhang

**Affiliations:** 1 State Key Laboratory of Subtropical Building Science, South China University of Technology, Guangzhou 510640, China; zhanghy@scut.edu.cn (H.Z.); 201821008854@mail.scut.edu.cn (J.L.); 2 Guangzhou Libi Real Estate Development Co., Ltd, Guangzhou 511399, China; 13424024950@163.com

**Keywords:** geopolymer concrete, demolished concrete lumps, axial compression test, scanning electron microscope, compressive strength

## Abstract

To reduce the environmental pollution from cement production and the damage to natural resources from aggregate mining in the concrete industry, a relatively new concrete, termed geopolymer recycled lump concrete (GRLC), which uses geopolymer as the binding material to replace traditional cement and uses large demolished concrete lumps (DCLs) to partly replace concrete, is prepared in this study. Cubic and cylindrical GRLC specimens containing fresh geopolymer concrete and DCLs were tested under axial compression with various parameters, including the compressive strength levels of both fresh geopolymer concrete and DCLs, and the replacement ratio of DCLs. The compressive behavior of the GRLC specimens was compared with traditional cement recycled lump concrete (CRLC) specimens, with test results showing that GRLC specimens possess higher compressive strength than CRLC specimens under the same experimental conditions, which is due to the strengthening effect that fresh geopolymer concrete has on the DCLs. From the scanning electron microscope pattern of the GRLC specimen, it is found that the geopolymer bonds well with the old mortar attached to DCLs. As the replacement ratio increases from 0% to 33%, the elastic modulus of GRLC increases by 5%–11% but Poisson’s ratio remains almost constant (in the 0.16–0.17 range). Based on the measured strength and the predicted results, which coincide with one another well, a modified method for predicting the compressive strength of GRLC cubic and cylindrical specimens is proposed.

## 1. Introduction

A large amount of solid waste is produced each year due to industrial production and structural dismantling. These solid wastes have a significant adverse environmental impact, such as occupying landfill space and contaminating water sources and air. To alleviate the adverse impact of solid wastes on the environment, the recycling of solid wastes in construction material has been extensively studied. For example, the reuse of various solid wastes in concrete [1,2], brick [3] and asphalt [4,5] has been reported, and the test results showed that many kinds of solid wastes can be reused in construction materials.

With the mass expansion of cities and the rapid development of infrastructure construction in developing countries like China, the demand for concrete has witnessed a dramatic increase in the last few decades. Accordingly, the consumption of natural aggregates and Portland cement, as the main components of ordinary concrete, has also increased substantially. Cement production releases a great amount of greenhouse gases and dust, bringing serious air pollution; based on production data in 2016 [6], annual cement production is responsible for about 8% of global annual CO_2_ emissions. The huge consumption of natural aggregates also damages the ecological environment and leads to the exhaustion of natural sand and stone resources. For the aforementioned issues, one possible solution is the replacement of Portland cement by green binder material and the recycling of solid waste into concrete for concrete production.

Geopolymer, an environmentally friendly green binding material, is considered as a promising alternative to Portland cement by many researchers [7,8,9]. It has comparable engineering properties as Portland cement, better fire resistance and durability, but emits less CO_2_ and consumes less energy during its production [10]. Sofi et al. [11] compared the compressive, splitting tensile and flexural strengths, modulus of elasticity, Poisson’s ratio of fly ash-based geopolymer concrete (GC) prepared by three different sources of Class-F fly ash, and found that GC has favorable engineering properties as ordinary Portland cement (OPC) concrete. Olivi et al. [12] reported that under similar compressive strength level, fly ash-based GC had higher flexural and tensile strengths, better durability in a seawater environment and less drying shrinkage than OPC concrete, but that the modulus of elasticity in GC was 15%–29% lower than that of OPC concrete. The experimental results from Zhang et al. [13] showed that geopolymer mortar exhibits similar bonding strength with commercial polymer cement mortar in a 25–700 °C temperature range. Mehta et al. [14] indicate that the higher compressive strength in fly ash-based GC is facilitated by its compact microstructure and better interfacial adhesion with aggregates.

**Figure 1:**
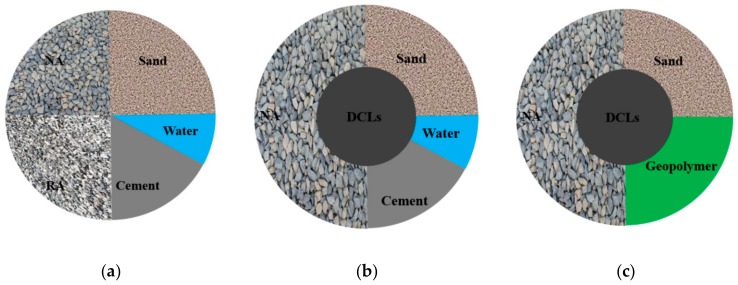
Concept of recycled aggregate concrete and recycled lump concrete. (**a**) Recycled aggregate concrete. (**b**) Cement recycled lump concrete. (**c**) Geopolymer recycled lump concrete.

Demolished concrete is a kind of common solid waste. As for the recycling of demolished concrete in concrete production, according to the literature, there are two different ways to do it: recycled aggregate concrete [15] and recycled lump concrete [16]. When demolished, concrete is crushed into particles with sizes smaller than 31.5 mm; the produced particles are called recycled aggregate (RA). Partly replacing natural aggregate (NA) in concrete with RA, as illustrated in Figure 1a, forms recycled aggregate concrete. Recycled lump concrete, on the other hand, is formed through crushing the demolished concrete into lump sizes larger than 60 mm, and then mixing the lumps with fresh concrete directly [17] to partly replace concrete in the process of concrete production, as shown in Figure 1b. Compared to RA, the energy consumption during the production of demolished concrete lumps (DCLs) is much less [18], and the contents of other waste (masonry, asphalt, glass, wood, etc.) are much lower. In addition, less cement is required for preparing recycled lump concrete than that for preparing recycled aggregate concrete with an equivalent total mass and equivalent mass of demolished concrete (recycled aggregates or lumps), since partial concrete is replaced by DCLs in recycled lump concrete. As a result, lower hydration heat is released during recycled lump concrete production. A series of studies showed that recycled lump concrete has comparable mechanical properties such as compressive strength and elastic modulus [19,20,21,22], tensile strength [23] and durability [24,25] with OPC concrete. The structural members, made of recycled lump concrete, exhibit similar flexural and shear behavior [26,27], compression behavior [28], fire resistance [29], seismic performance [30] and long-term creep behavior [31] as the benchmark members made with fresh OPC concrete alone. In China, the successful application of recycled lump concrete to concrete beams, slabs and columns has been seen in several multi-story buildings [32,33,34].

When the binding material in recycled aggregate concrete (Portland cement) is replaced by a geopolymer, the produced concrete is referred to as geopolymer recycled aggregate concrete. A few researchers have investigated the mechanical properties of geopolymer recycled aggregate concrete. Nuaklong et al. [35] found that the average compressive strength of geopolymer recycled aggregate concrete, with a replacement ratio of 100%, is about 13% lower than that of fresh pure GC. Shaikh [36] reported that the compressive strength of geopolymer recycled aggregate concrete having a 50% replacement ratio, at 28-day curing age, decreased by 18.7% compared to that without RA, and that the extent of the decrease increased as the replacement ratio of RA increases.

If a geopolymer is substituted for Portland cement as the binding material in recycled lump concrete, a new type of concrete is derived, termed ‘geopolymer recycled lump concrete (GRLC)’, as seen in Figure 1c. In comparison with ordinary recycled lump concrete, the energy consumption and carbon emissions during the production of GRLC are much less. However, no experimental data on the mechanical properties of GRLC have been reported in the literature.

In this study, the mechanical behavior of GRLC was investigated through cylindrical and cubic specimens under axial compression. The effects of replacement ratio, strength levels of DCLs and fresh GC on the compressive strength, elastic modulus, Poisson’s ratio and volumetric strain of GRLC were evaluated. The shape effect and size effect of GRLC specimens were also analyzed. Utilizing the experimental results obtained from the current study, a formula for predicting the compressive strength of GRLC is proposed. Based on the test results, it can be concluded that GRLC specimens possess better compressive performance than ordinary recycled lump concrete specimens. Therefore, GRLC may be used as a substitute for ordinary concrete in the future, with the application in similar engineering fields as that of ordinary recycled lump concrete.

## 2. Experimental Program

### 2.1. Materials and Mix

To prepare the geopolymer binder, a blend of fly ash (FA) and metakaolin (MK), with a mass ratio of 1:1, is activated by potassium silicate solution with molar ratio (K_2_O/SiO_2_) = 1 and a mass concentration of 40%. Commercially produced metakaolin with an average particle size of 0.017 mm and a density of 2530 kg/m^3^, and low calcium fly ash with an average particle size of 0.032 mm and a density of 2620 kg/m^3^, were sourced from suppliers in China. Table 1 lists the chemical compositions of FA and MK, obtained through X-ray fluorescence analysis.

Two batches of demolished concrete, with different strength levels, were crushed into two types of DCLs, respectively (named D1 and D2, with a corresponding density of 2430 kg/m^3^ and 2470 kg/m^3^), with a particle size of 70–100 mm. The compressive strength of the two types of demolished concrete, measured by Φ100 × 100 mm cylinder core drilling samples, were 35.7 MPa and 51.6 MPa, respectively. The corresponding 150 × 150 × 150 mm cubic compressive strength was 35.7 MPa and 51.6 MPa, calculated through the relationship between cylindrical and cubic compressive strength of OPC concrete [37]. Three types of fresh concrete, OPC, GC1 and GC2, were mixed with DCLs to derive recycled lump concrete. The natural coarse aggregate with a maximum particle size of 20 mm and density of 2650 kg/m^3^, and local river sand with a fineness modulus of 2.7 and density of 2580 kg/m^3^, were used in fresh concrete. Table 2 and Table 3 present the mix proportions and compressive strengths of the three types of fresh concrete, in which the compressive strength was tested through Φ150 × 300 mm cylinders and 150 × 150 × 150 mm cubes, respectively.

### 2.2. Specimen Design and Preparation

Due to the large size of DCLs (70–100 mm), the cylindrical and cubic specimens with a characteristic size (the minimum size in a specimen) of 300 mm, namely Φ300 × 600 mm cylinders and 300 × 300 × 300 mm cubes, were used for axial compression tests on recycled lump concrete. Another test variable is the replacement ratio *η* of DCLs, which is defined as the weight ratio of DCLs in the specimen to the whole specimen. Totally, thirty-three 300 × 300 × 300 mm cubic specimens and twenty-four Φ300 × 600 mm cylindrical specimens divided into nineteen groups (i.e., three identical specimens in each group) were fabricated by mixing three types of fresh concrete with two types of DCLs. Table 4 lists the details of these specimens. The designation of specimens consisted of the type of fresh concrete (GC1, GC2, OPC), type of DCLs (D1, D2), replacement ratio *η* of DCLs (20% and 33%) and the shape of the specimens (“Y” and “U” is for cylinder and cube, respectively). For example, “GC1-D2-33-Y” represents a cylindrical specimen made of GC1 fresh concrete and D2 demolished lumps, with a replacement ratio of 33%.

**Figure 2:**
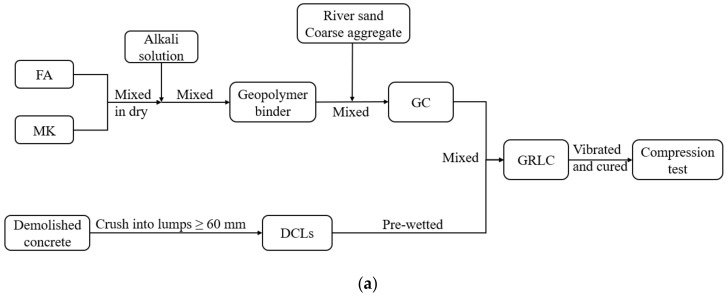

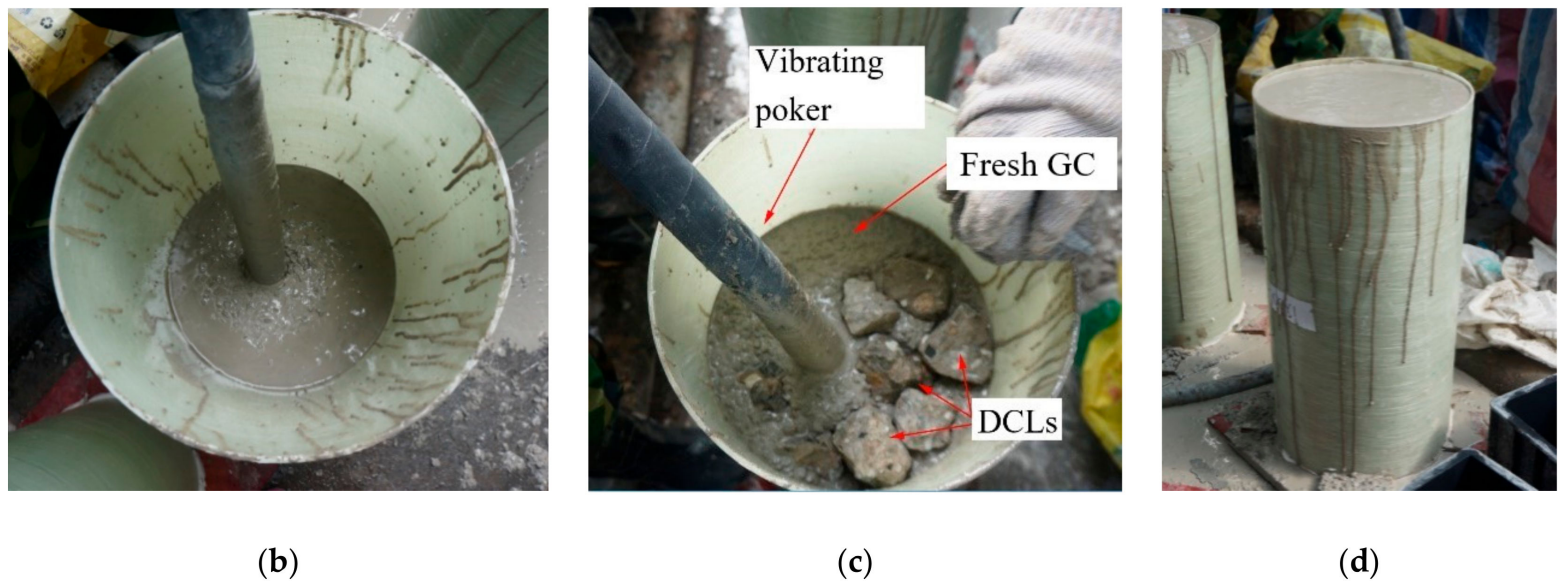
Preparation of geopolymer recycled lump concrete (GRLC) specimens, (**a**) Preparation process flowchart of GRLC specimens. (**b**) Fresh geopolymer concrete (GC) layer. (**c**) Vibration. (**d**) Specimens molding.

Since the water absorption of DCLs is relatively high (5.66% and 3.41% for D1 and D2, respectively), the DCLs were wetted before pouring into the mold in order to avoid the water in fresh concrete being absorbed by the DCLs. During casting, a layer of 20 mm thickness fresh concrete was first poured into the mold, then the DCLs and fresh concrete were added alternately. To evenly distribute the DCLs in the mixture, a vibrating poker was used to stir the mixture of DCLs and fresh concrete. After casting, specimens were covered with sacks and then cured at room temperature for 28 days prior to undertaking compression tests. The preparation process of GRLC specimens is illustrated in Figure 2.

### 2.3. Testing Procedures and Instruments

Axial compression tests were conducted on GRLC and cement recycled lump concrete (CRLC) specimens through an electro-hydraulic loading machine with a capacity of 10,000 kN. The top and bottom surfaces of each specimen were coated with high-strength gypsum to ensure that the two loading surfaces were horizontal. The axial load was applied at a strain rate of 10^−5^/s as specified in the ASTM C39 standard [38]. The deformation and strain data were collected synchronously by a JM 3813 multifunctional acquisition system.

**Figure 3:**
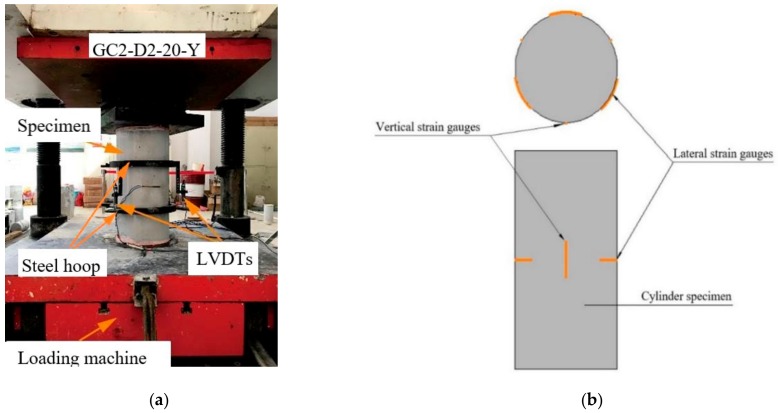
Test set-up and location of strain gauges. (**a**) Test set-up. (**b**) Locations of strain gauges.

Cubic specimens were only tested for the compressive strength of specimens, but cylindrical specimens undertook the measurement of the compressive strength, Poisson’s ratio, elastic modulus and volumetric strain. Therefore, three linear variable differential transducers (LVDTs), with a ±5 mm calibration range, were vertically fixed through two steel hoops around the periphery of the cylinders, to measure the axial deformations in the middle 2/5-height of the cylinders (see Figure 3a). To measure vertical and lateral strain in the concrete, three vertical strain gauges and three lateral strain gauges were mounted at the mid-height of specimens with equal spacing, as shown in Figure 3b.

After axial compression tests, scanning electron microscope (SEM) analyses were carried out through Zeiss EVO 18 to investigate the microstructure of several GRLC specimens. Three 5 × 5 mm slices, with an interface transition zone between fresh GC and DCLs included, were taken from each broken specimen. The surfaces of these slices were coated with a thin layer of gold using a sputter-type coater to obtain clear images for SEM analysis [39].

## 3. Results and Discussion

### 3.1. Failure Pattern

**Figure 4:**
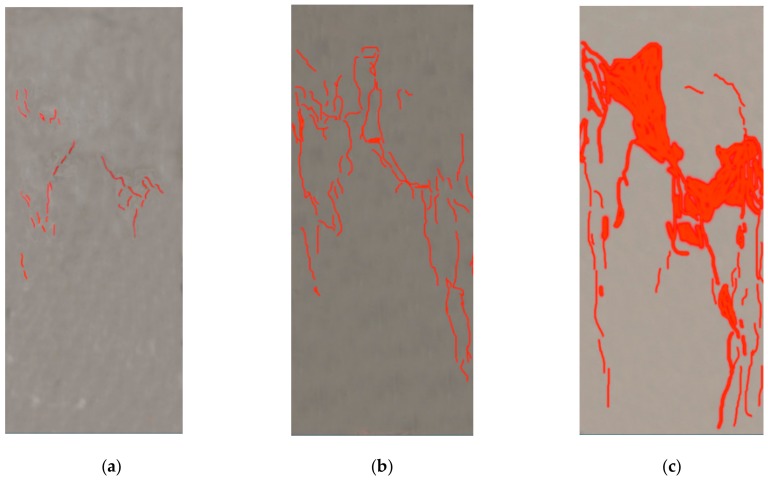
Crack development of specimen GC2-D1-33-Y. (**a**) Initial cracks. (**b**) At the peak load. (**c**) After the peak load.

**Figure 5:**
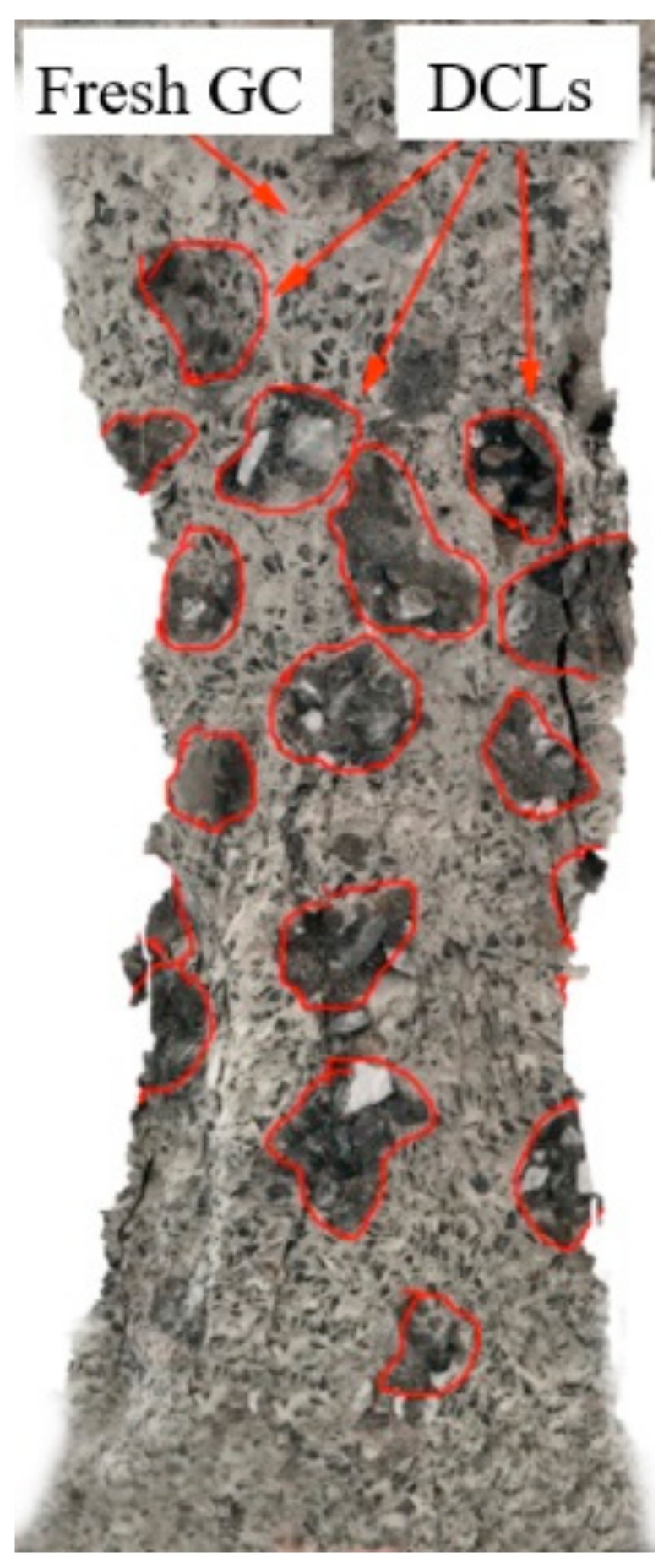
Failure pattern in specimen GC2-D1-33-Y.

Under axial compression, crack development in the GRLC specimens was similar to that of GC specimens without DCLs. Until loading to about 80% of the peak load, no noticeable cracks formed on the surfaces of the specimens. With a further increase in the applied load, cracks appeared and quickly propagated. When the applied load attained the peak value, cracks almost ran through the whole height of the specimen. After the peak value, although the load decreased, the axial deformation (strain) increased, and diagonal main cracks formed. Finally, the concrete near the mid-height bulged out and fell-off, and the specimen failed. Figure 4 and Figure 5 present the crack development process and failure pattern, respectively, in specimen GC2-D1-33-Y. The cracks on these figures were sketched using Photoshop software by a transparent layer on the pictures taken by a HD camera during the compression test, which is a rough method to outline the development and width of cracks. The digital image correlation (DIC) technology is a more accurate method to detect and describe cracks [40,41], but unfortunately it was not used in this study. As shown in Figure 5, the failure surface ran through the fresh geopolymer concrete and many DCLs, but not along the interface between DCLs and fresh GC. It can therefore be inferred that the interface between DCLs and fresh GC is not the weak part in GRLC. To verify this inference, SEM analysis was conducted after the axial compression test to examine the microstructure of GRLC.

**Figure 6:**
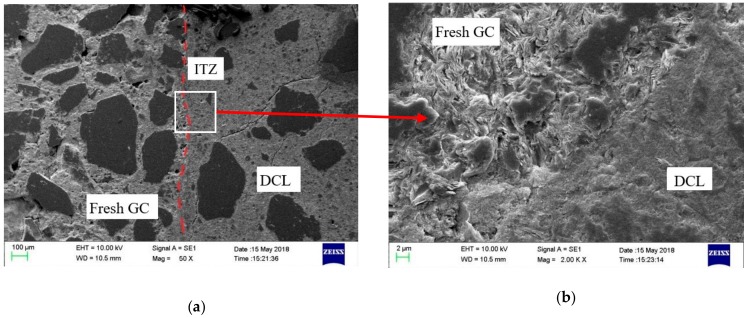
SEM pattern of specimen GC2-D1-33-Y. (**a**) Magnification of 50 times. (**b**) Magnification of 2000 times.

Figure 6 presents the SEM pattern of specimen GC2-D1-33-Y. From Figure 6a, the interface transition zone (ITZ) between fresh GC and DCL can be clearly seen, but no noticeable cracks are observed at the ITZ. Magnifying the ITZ between the fresh GC and DCL, it can be observed that the geopolymer was well bonded with the mortar attached to DCLs, as shown in Figure 6b. Due to the lower strength of DCLs, several cracks were seen in the old mortar and ITZ between coarse aggerate and old mortar in DCLs. These cracks may be formed during the crushing of demolished concrete or during the compression test.

### 3.2. Compressive Strength

#### 3.2.1. Influencing Factor

**Figure 7:**
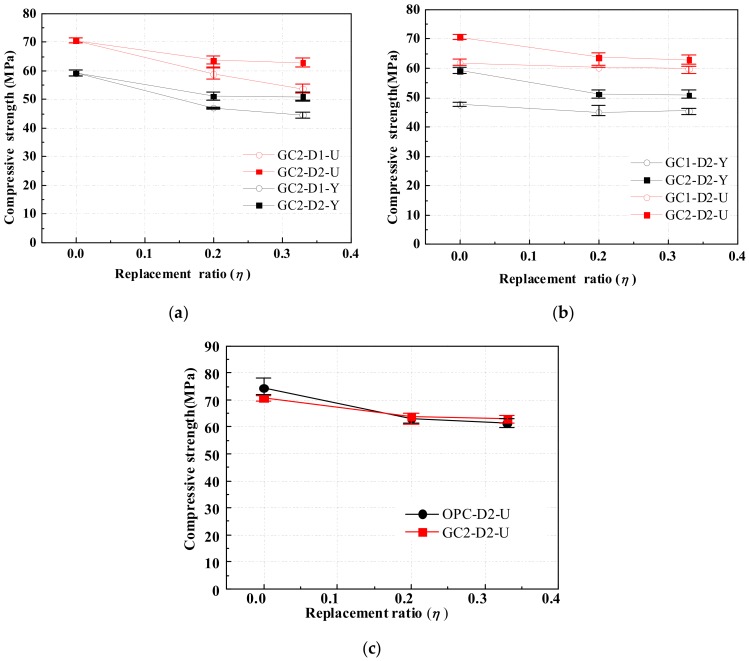
Effect of strength levels of demolished concrete lumps (DCLs) and fresh concrete, and type of fresh concrete on compressive strength of RLC. (**a**) Different strength levels of DCLs. (**b**) Different strength level of fresh GC. (**c**) Different types of fresh concrete.

The cubic and cylindrical compressive strength of GRLC specimens and cubic compressive strength of CRLC specimens were measured. As a function of the replacement ratio, Figure 7a,b, respectively, show the compressive strength of GRLC specimens with the same strength levels of fresh GC but different strength levels of DCLs, and specimens with the same strength levels of DCLs but different strength levels of fresh GC. As illustrated in Figure 7, the compressive strength of GRLC decreases with a decrease in the strength of fresh GC and DCLs and an increase in the replacement ratio of DCLs. However, in cases where the difference in strength between fresh and demolished concrete is lower, the effect of the replacement ratio on the compressive strength of GRLC is less significant. For instance, the difference between fresh GC1 and recycled D2 in compressive strength is 13.2 MPa. When the weight ratio of D2 in the specimen is increased from 20% up to 33%, the compressive strength of specimens in Group GC1-D2-Y almost had no reduction and that of specimens in Group GC1-D2-U only decreased by 1.6%. However, such reduction in compressive strength of the specimens in Group GC2-D1-Y (U), with the strength difference in fresh and demolished concrete being 38.3 MPa, is 5.3% and 8.6% for specimens in Groups GC2-D1-Y and GC2-D1-U, respectively. This is due to that when demolished concrete has a much lower strength than that of fresh concrete, more microstructural flaws or weak points exist in the demolished concrete, leading to a higher possibility of DCLs being crushed under axial compression at a higher replacement ratio. Combining Figure 7a,b, it can be observed that the variation in compressive strength of the GRLC cylindrical specimen with the replacement ratio of DCLs is similar to that of its counterpart cubic specimen. This is not in line with the trend in CRLC specimens reported by Wu et al. [21] who found from their study that the replacement ratio of DCLs has a more significant influence on the compressive strength of CRLC cylindrical specimens than on cubic specimens.

Figure 7c compares the variation in compressive strength of GRLC specimens (GC2-D2-U) and CRLC specimens (OPC-D2-U) with replacement ratio. The DCLs used in the two groups of specimens were similar but the strength level of fresh concrete in Group OPC-D2-U was slightly higher than that in GC2-D2-U. As can be seen from Figure 7c, the compressive strength in the two groups of specimens both decreased with an increasing replacement ratio of DCLs, but the variation in GRLC specimens is at a slower rate. Especially, at a replacement ratio of 20% and 33%, the compressive strength of specimens in Group GC2-D2-U, having lower strength level in fresh concrete, is even slightly higher than that of OPC-D2-U.

**Figure 8:**
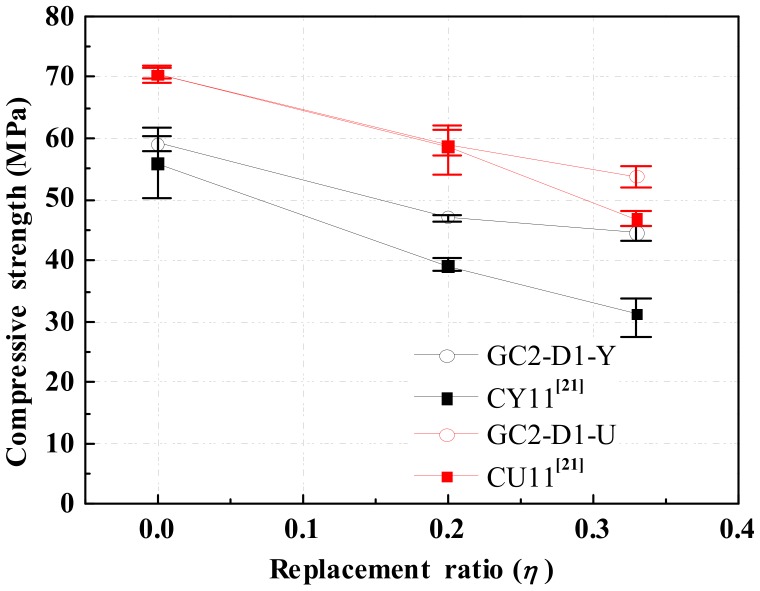
Comparison on compressive strength of GRLC specimens in this study and cement recycled lump concrete (CRLC) specimens in Ref. [21].

Figure 8 compares the compressive strength of GRLC measured through cubic and cylindrical specimens in the current study, and that of CRLC specimens from Ref. [21] having similar strength levels of fresh concrete and DCLs (74.9 MPa and 33.1 MPa, respectively) as the GRLC specimens in the current study. A similar trend, i.e., the effect of replacement ratio on the compressive strength of GRLC specimens is less significant than that on the counterpart CRLC specimens, is seen.

Combining Figure 7c and Figure 8, it can be concluded that the fresh GC had a certain strengthening effect on the demolished concrete, which can be attributed to the following two reasons. Firstly, the potassium silicate component in the geopolymer mixture penetrates into the pores and microcracks in the DCLs during mixing and stirring of fresh GC and DCLs, and reacts with the residual calcium hydroxide in the old mortar in DCLs to form C–S–H gels [42], thereby filling and repairing the pores and microcracks in the DCLs. Besides, geopolymer mortar has better bonding properties than OPC mortar [43], which leads to better bonding at the interface between fresh GC and DCLs in GRLC specimens, as shown in Figure 6b.

#### 3.2.2. Shape Effect

**Figure 9:**
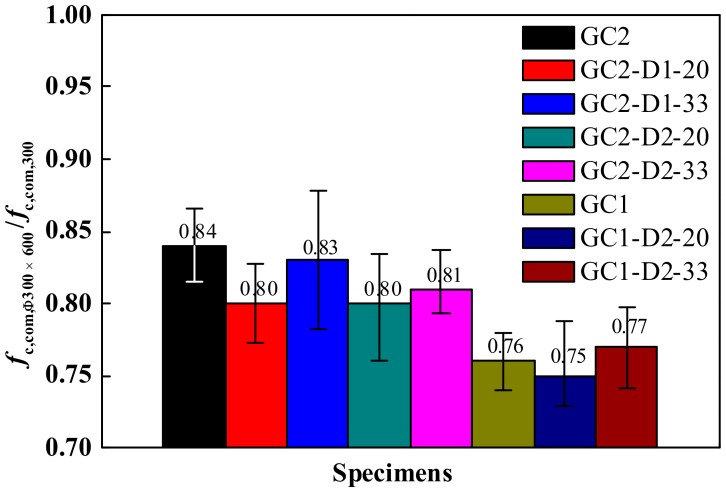
Ratio of compressive strength of GRLC cylindrical specimen to cubic specimen

The compressive strength ratio of the concrete cylindrical specimen to cubic specimen represents the shape effect on strength. Figure 9 plots the ratio of compressive strength of Φ300 × 600 mm GRLC cylindrical specimens to that of 300 × 300 × 300 mm GRLC cubic specimens. The ratio (mean value from three specimens in each group) was in the 0.75–0.84 range, which was mainly dependent on the strength of fresh concrete, but not greatly affected by the replacement ratio of DCLs. However, the corresponding ratio in CRLC specimens, with similar strength levels in fresh and demolished concrete, ranged from 0.61 to 0.69 as reported in Ref. [21]. The higher ratio in compressive strength of cylindrical to cube specimens of GRLC implies that the shape effect of GRLC on compressive strength was less significant than that of CRLC.

#### 3.2.3. Prediction Formula

To predict the compressive strength of CRLC specimens, Wu et al. [21] proposed the following formula, based on three batches of experimental data:

(1) $$f_{\mathrm{cu},\mathrm{com},300}={\left(\frac{f_{\mathrm{cu},\mathrm{old},300}}{f_{\mathrm{cu},\mathrm{new},300}}\right)}^{0.86\eta }\times f_{\mathrm{cu},\mathrm{new},300}\times \left(1-\eta \right)+{\left(\frac{f_{\mathrm{cu},\mathrm{new},300}}{f_{\mathrm{cu},\mathrm{old},300}}\right)}^{1.1\eta }\times f_{\mathrm{cu},\mathrm{old},300}\times \eta$$

in which *f*_cu,com,300_ denotes the compressive strength of 300 × 300 × 300 mm cubic CRLC specimens containing fresh concrete with old concrete, *f*_cu,new,300_ and *f*_cu,old,300_ represent the compressive strength of new (fresh) and old (recycled) concrete of 300 × 300 × 300 mm cubic specimens, respectively, and *η* is the replacement ratio of DCLs. On condition that the size effect on cubic compressive strength of fresh concrete, recycled concrete and CRLC are taken into account. Equation (1) can be modified to predict the cubic compressive strength of CRLC with other sizes, such as 150 × 150 × 150 mm cubic compressive strength [21].

When the strengths of new and old concrete are close, such as |*f*_cu,new,150_-*f*_cu,old,150_| ≤ 15 MPa [44], Equation (1) can be simplified to:

(2) $$f_{\mathrm{cu},\mathrm{com},300}=f_{\mathrm{cu},\mathrm{new},300}\times \left(1-\eta \right)+f_{\mathrm{cu},\mathrm{old},300}\times \eta$$

However, if *f*_cu,new,300_ is much higher than *f*_cu,old,300_ then the influence coefficient of *f*_cu,old,300_ is larger than 1.0 ((*f*_cu,new,300_/*f*_cu,old,300_)^1.1*η*^ > 1.0), and the influence coefficient of *f*_cu,new,300_ is smaller than 1.0 ((*f*_cu,new,300_/*f*_cu,old,300_)^0.86*η*^ < 1.0). This coincides with the observed trend that when the difference in strength between new and old concrete is higher, the influence of DCLs on the compressive strength of GRLC is more significant.

For CRLC cylindrical specimens, the two exponents in Equation (1) were changed from 0.86 *η* and 1.1 *η* to 2.2 *η* and 1.3 *η*, respectively, due to the fact that the compressive strength of demolished concrete has greater influence on the compressive strength of CRLC cylinders than on that of CRLC cubes [18].

Equations (1) and (2) were tentatively used for predicting the compressive strength of GRLC specimens. Since the effect of DCLs on the compressive strength of the GRLC cylindrical specimen is similar to that on the GRLC cubic specimen, the same exponents (0.86 *η* and 1.1 *η*) were adopted for GRLC cubic and cylindrical specimens. That is, the cubic compressive strengths (*f*_cu,new,300_, *f*_cu,old,300_ and *f*_cu,com,300_) in Equation (1) can be replaced by the cylindrical compressive strength of GRLC.

The compressive strengths of GRLC and CRLC were tested through 300 × 300 × 300 mm cubes or Φ300 × 600 mm cylinders in this study, but the compressive strengths of fresh and demolished concrete are generally tested through 150 × 150 × 150 mm cubic or Φ150 × 300 mm cylindrical specimens. Therefore, the compressive strengths tested through 150 × 150 × 150 mm cubic or Φ150 × 300 mm cylindrical specimens have to be converted to those of 300 × 300 × 300 mm cubes or Φ300 × 600 mm cylinders before substituting into Equation (1). For normal-strength Portland cement concrete, the ratio of the compressive strength of specimens with a characteristic size of 300 mm to that of 150 mm can be taken as 0.87 (*f*_cu,300_/*f*_cu,150_) and 0.83 (*f*_c,Φ300×600_/*f*_c,Φ150×300_) for cube and cylinder, respectively, as reported in Ref. [44] and Ref. [20]. In case of fresh GC, this ratio can be taken as 0.95 (*f*_cu,300_/*f*_cu,150_) and 0.91 (*f*_c,Φ300×600_/*f*_c,Φ150×300_) for cubic and cylindrical specimens, respectively, based on the mean experimental results of GC1 and GC2 specimens (see Table 3 and Table 4).

**Figure 10:**
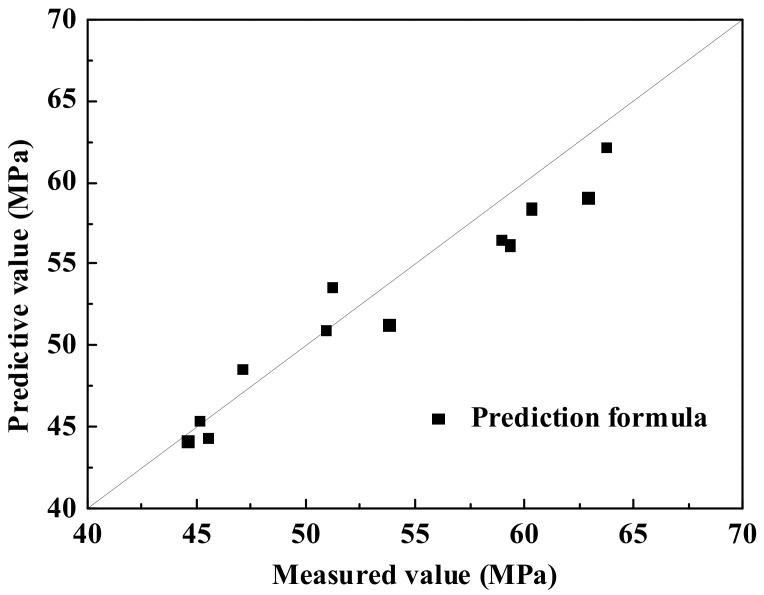
Comparison between predicted and measured compressive strength of GRLC specimens.

Since the geopolymer has a certain strengthening effect on DCLs, the application scope of the simplified formula, Equation (2), can be appropriately broadened to slightly reduce the negative impact of DCLs on the compressive strength of GRLC specimen. After trial calculation, it is found that when |*f*_cu,new,150_-*f*_cu,old,150_| ≤ 20 MPa, the simplified Equation (2) is applicable to predict the compressive strength of GRLC cubic and cylindrical specimens. In case of |*f*_cu,new,150_-*f*_cu,old,150_| > 20 MPa, the prediction result using Equation (1) agreed well with the measured results. A comparison of the predicted compressive strength of GRLC specimens using Equation (1) or (2) with the measured results is shown in Table 4 and Figure 10.

### 3.3. Elastic Modulus

Three LVDTs were installed around each cylindrical specimen to measure the vertical deformation, from which the vertical strain was calculated, and then the elastic modulus of GRLC specimens was determined [45].

**Figure 11:**
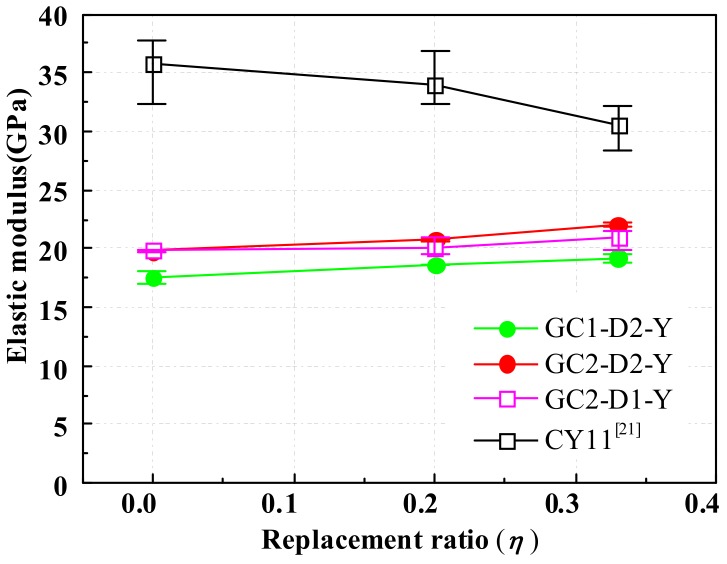
Elastic modulus of GRLC and CRLC specimens.

Figure 11 presents the elastic modulus of GRLC cylinders with a different a replacement ratio of DCLs. The elastic modulus of CRLC specimens, reported by Ref. [21], were also plotted in Figure 11 for comparison. As elucidated in the figure, GRLC specimens exhibit much lower elastic modulus than CRLC specimens. This is due to the lower elastic modulus being of geopolymer concrete than that of OPC concrete. There are experimental studies showing that the elastic modulus of fresh GC is about 67%–73% of that of OPC concrete under the same strength level [46,47]. In this study, the elastic modulus of fresh GC1 and GC2 is only 17.5 GPa and 19.8 GPa, respectively, which is about 55% of that of fresh OPC concrete in Ref. [21] (35.7 GPa). In addition, it can be seen from Figure 11 that the elastic modulus of GRLC specimens increases slightly (5%–11%) but that of CRLC decreases gradually, with the replacement ratio of DCLs increasing from 0% to 33%. This also results from the lower elastic modulus of fresh geopolymer concrete than that of demolished OPC concrete in GRLC. Therefore, the addition of DCLs increases the elastic modulus of GRLC, and the greater the amount of incorporated DCLs, the greater the increment of elastic modulus in GRLC, as compared to geopolymer concrete without DCLs.

### 3.4. Poisson’s Ratio

Three vertical and three lateral strain gauges were mounted at mid-height around each cylindrical specimen. The data measured by these strain gauges, at a loading ratio *λ* (the ratio of applied load to the peak load) of 0.4, were utilized to calculate Poisson’s ratio of GRLC specimens.

**Figure 12:**
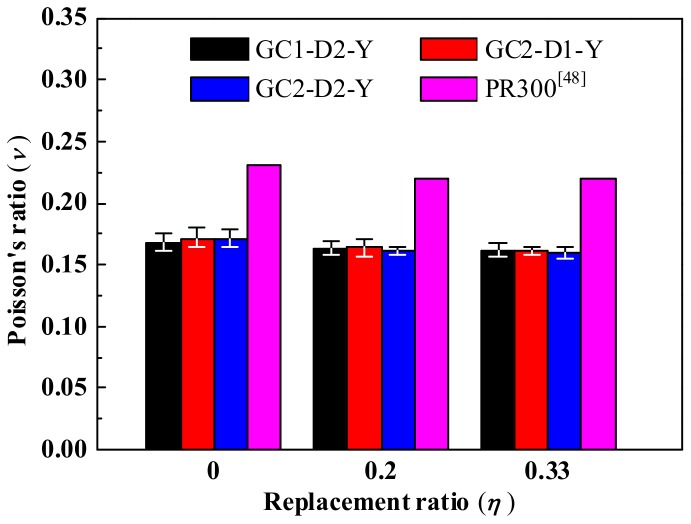
Poisson’s ratio of GRLC specimens and cement compound concrete in Ref. [48] (*λ* = 0.4).

Figure 12 shows Poisson’s ratio of GRLC cylinders obtained in the current study and CRLC cylinders reported in Ref. [48], as a function of replacement ratio. It can be observed that the strength level of fresh GC and demolished OPC concrete, and the replacement ratio of DCLs, almost has no influence on Poisson’s ratio *υ* of GRLC. Poisson’s ratio of GRLC is about 0.16–0.17, which is in the range of conventional OPC concrete (0.14–0.26) suggested in the CEB-FIP Model 2010 [49]. Compared to the CRLC reported in Ref. [48], Poisson’s ratio of GRLC in the current study was about 65% of that of the former at the same replacement ratio.

### 3.5. Volumetric Strain

Under axial compression, concrete experiences axial compaction and then lateral expansion, thus the volume of concrete changes. Volumetric strain, defined as *ε*_vol_ = *ε*_1_ + 2*ε*_2_, where *ε*_1_ is the vertical strain of the concrete (to be a positive value), and *ε*_2_ is the lateral strain of the concrete (to be a negative value), is often adopted to represent the volume variation of concrete under axial compression.

**Figure 13:**
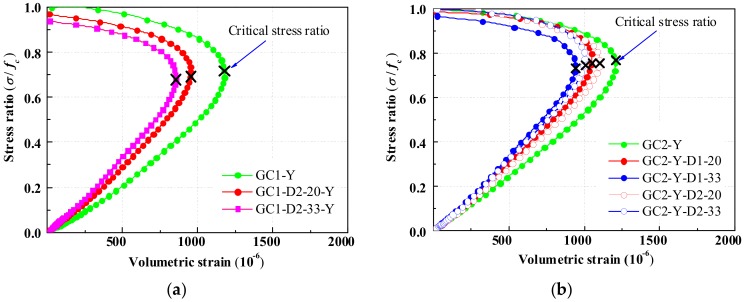
Stress–volumetric strain curve of cylindrical specimens. (**a**) Fresh geopolymer concrete of GC1. (**b**) Fresh geopolymer concrete of GC2.

The volumetric strain curves of GRLC cylinders, using GC1 and GC2 as fresh concrete, were plotted in Figure 13a,b, respectively. With an increase in axial stress ratio *σ*/*f*_c_ (the ratio of axial stress to compressive strength), the volumetric strain increases gradually and then turns to decrease until failure. This implies that the volume of the concrete shrinks first and then expands. The stress ratio *σ*/*f*_c_ corresponding to the turning point on the curve, called the critical stress ratio, represents the stress ratio at which the concrete volume begins to expand but not continuing to shrink.

As seen in Figure 13a, the stress–volumetric strain curves of GRLC specimens with different replacement ratios are almost coincident when the stress ratio is lower than 0.1. However, with a further increase in the stress ratio, a significant difference occurs in the volumetric strains of specimens with different replacement ratios. The specimen with a higher replacement ratio experiences lower volumetric strain under the same stress ratio. This is because the elastic modulus of cylinders increased with the replacement ratio of DCLs, as stated in Section 3.3, which led to lower vertical strain and thus lower volumetric strains in GRLC specimens with a higher content of DCLs under the same stress level.

From Figure 13b, the critical stress ratio of specimens decreased with a decrease in compressive strength of DCLs or an increase in replacement ratio. This implies that under axial compression, the volume of GRLC specimens with lower compressive strength or higher content of DCLs began to expand under a lower stress level [50]. This is mainly due to more flaws (such as microcracks) present in the DCLs in that case.

Comparing Figure 13a,b, it can be seen that the critical stress ratio of specimens with a higher strength level in fresh GC is higher than that of specimens with lower strength level in fresh GC. This is facilitated by the better bonding between fresh concrete and DCLs when using higher strength fresh GC to mix with DCLs.

## 4. Conclusions

Axial compression tests were carried out on a great amount of geopolymer recycled lump concrete (GRLC) cubic and cylindrical specimens with different replacement ratios of recycled concrete lumps (DCLs) and strength levels in DCLs and fresh geopolymer concrete. Based on the experimental data, the fundamental mechanical properties of GRLC were evaluated and the following conclusions were drawn.

1. Geopolymer concrete is well bonded with the old mortar in DCLs and has a certain strengthening effect on DCLs. Under similar conditions, the compressive strength of GRLC specimens is higher than that of ordinary cement recycled lump concrete (CRLC), but the shape effect of GRLC specimens is less significant than the latter.
2. After a minor modification, the formula for predicting the cubic compressive strength of CRLC is applicable to predict the compressive strength of GRLC cubic and cylindrical specimens.
3. With the replacement ratio of DCLs increasing, the elastic modulus of the GRLC specimens increases but Poisson’s ratio is kept in the 0.16–0.17 range.
4. The critical stress ratio, which represents the stress level at which the volume of concrete begins to expand under axial compression, decreases with a decrease in the strength of DCLs or an increase in the replacement ratio of DCLs in GRLC specimens.

In general, GRLC has comparable compressive performance, but lower energy consumption and carbon emissions during its production than that of CRLC. The latter has been used in practical construction. With the increasing concern to environmental protection, the use of GRLC in practical construction, as a promising substitution of ordinary concrete, is possible in the foreseeable future.

## Figures and Tables

**Table 1:** Chemical composition (wt%) of fly ash and metakaolin.

Material	SiO_2_	Al_2_O_3_	CaO	Fe_2_O_3_	TiO_2_	SO_3_	MgO	K_2_O	P_2_O_5_	Others	Loss on Ignition
FA	51.35	44.24	0.13	0.98	0.90	0.00	0.48	0.08	0.45	0.17	0.72
MK	45.3	41.2	3.77	3.18	1.62	0.75	0.44	0.38	0.36	0.33	2.40

**Table 2:** Mix proportions and compressive strength of fresh cement concrete.

Type of Fresh Concrete	Water (kg/m^3^)	Cement kg/m^3^	Sand (kg/m^3^)	Coarse Aggregate (kg/m^3^)	Superplasticizer (kg/m^3^)	Cubic Compressive Strength (MPa)
OPC	175	547	611	1242	5.47	82.5

**Table 3:** Mix proportions and compressive strength of fresh geopolymer concrete.

Type of Fresh Concrete	Water (kg/m^3^)	MK (kg/m^3^)	FA (kg/m^3^)	Sand (kg/m^3^)	Coarse Aggregate (kg/m^3^)	KOH (kg/m^3^)	Potassium Silicate (kg/m^3^)	Cubic Compressive Strength (MPa)	Cylindrical Compressive Strength (MPa)
GC1	70	177	177	529	1236	68	244	64.8	47.7
GC2	63	186	186	535	1249	61	219	74.0	59.2

**Table 4:** Details of specimens.

Group	Type of Fresh Concrete	Type of Demolished Concrete	*η* (%)	Specimen Shape	*f*_cu,new,150_ (MPa)	*f*_cu,old,150_ (MPa)	*f*_c, com,300_ (MPa)	
							Measured	Calculated
GC1-U	GC1	--	--	Cube	64.8	--	61.8	--
GC2-U	GC2	--	--	Cube	74.0	--	70.6	--
OPC-U	OPC	--	--	Cube	82.5	--	74.4	--
GC1-D2-20-U	GC1	D2	20	Cube	64.8	51.6	60.3	58.4
GC1-D2-33-U	GC1	D2	33	Cube	64.8	51.6	59.3	56.2
GC2-D1-20-U	GC2	D1	20	Cube	74.0	35.7	58.9	56.5
GC2-D1-33-U	GC2	D1	33	Cube	74.0	35.7	53.8	51.3
GC2-D2-20-U	GC2	D2	20	Cube	74.0	51.6	63.7	62.2
GC2-D2-33-U	GC2	D2	33	Cube	74.0	51.6	62.9	59.1
OPC-D2-20-U	OPC	D2	20	Cube	82.5	51.6	63.0	--
OPC-D2-33-U	OPC	D2	33	Cube	82.5	51.6	61.4	--
GC1-Y	GC1	--	--	Cylinder	64.8	--	47.7	--
GC2-Y	GC2	--	--	Cylinder	74.0	--	59.2	--
GC1-D2-20-Y	GC1	D2	20	Cylinder	64.8	51.6	45.1	45.4
GC1-D2-33-Y	GC1	D2	33	Cylinder	64.8	51.6	45.5	44.4
GC2-D1-20-Y	GC2	D1	20	Cylinder	74.0	35.7	47.1	48.6
GC2-D1-33-Y	GC2	D1	33	Cylinder	74.0	35.7	44.6	44.2
GC2-D2-20-Y	GC2	D2	20	Cylinder	74.0	51.6	51.2	53.6
GC2-D2-33-Y	GC2	D2	33	Cylinder	74.0	51.6	50.9	51.0

Note: *f*_cu,new,150_ and *f*_cu,old,150_ is 150 × 150 × 150 mm cubic compressive strength of fresh concrete and demolished concrete on the test day, respectively; and *f*_c, com,300_ is the combined compressive strength of recycled lump concrete (cubic or cylindrical) specimens with a characteristic size of 300 mm on the test day.
